# Discontinuation of treatment of schizophrenic patients is driven by poor symptom response: a pooled post-hoc analysis of four atypical antipsychotic drugs

**DOI:** 10.1186/1741-7015-3-21

**Published:** 2005-12-23

**Authors:** Hong Liu-Seifert, David H Adams, Bruce J Kinon

**Affiliations:** 1 Lilly Research Laboratories, Eli Lilly and Company, Indianapolis, Indiana, USA

## Abstract

**Background:**

Stopping antipsychotic treatment can interrupt improvement and exacerbate the illness. The reasons for discontinuing treatment during controlled clinical trials were analyzed to explore this phenomenon.

**Methods:**

A post-hoc, pooled analysis was made of 4 randomized, double-blind clinical trials, 24–28 weeks in duration, involving 1627 patients with schizophrenia or a related disorder. Analyses combined all the atypical antipsychotic treatment groups in the studies.

**Results:**

The majority of patients (53%) stopped their treatment at an early stage. Poor psychiatric response along with worsening symptoms was the most frequently given reason for discontinuing the course (36%), which was substantially more common than discontinuation due to poor tolerability of the medication (12%). This phenomenon was corroborated by less improvement in patients who discontinued treatment compared with those who completed, based on the PANSS total scores. Discontinuation due to poor response was, apparently, more predominantly linked to patient perception than to physicians' conclusions alone (80% vs. 20%). Discontinuation due to patient perception of poor response appeared to occur particularly early in the course of treatment. Patients who discontinued due to poor toleration of the medication responded in a more comparable manner with completers.

**Conclusion:**

Discontinuing treatment may lead to exacerbation of symptoms, undermining therapeutic progress. In these studies, poor response to treatment and worsening of underlying psychiatric symptoms, and to a lesser extent, intolerability to medication were the primary contributors to treatment being discontinued. Our findings suggest that adherence may be enhanced by effective symptom control, as objectively measured and as subjectively perceived. Such strategies may improve patients' willingness to undertake long-term therapy and increase the likelihood of a better prognosis.

## Background

Adherence to a drug regime is a significant issue in the clinical management of schizophrenia. Early treatment discontinuation on the part of patients with schizophrenia or schizophrenia-like disorders is strikingly common, with estimates of its prevalence in antipsychotic drug trials ranging from 25%–75%. The rates of nonadherence appear to be even higher in natural, uncontrolled settings [1-4]. The consequences of early termination of the treatment are significant, making adherence to medication a critical determinant of a generally good prognosis. Discontinuing a prescribed antipsychotic drug is associated with symptom exacerbation [5], relapse [5,6], increased hospitalization [5,6], poor long-term course of illness [7], and higher economic costs of treatment [8]. Seventy-five per cent of patients who stop taking their antipsychotic medication experience significant worsening of symptoms over the course of a year compared with only 25% of those who consistently take their medication [5,6].

There are many factors associated with stopping treatment at an early stage. These can be separated into causes, such as:

• treatment-related reasons, e.g. inadequate response and adverse events;

• patient-related reasons, e.g. insight and attitude;

• and environmental elements, e.g. family support and transportation availability [5,9,10].

Adverse effects of treatment are one of the more frequently cited reasons for noncompliance with antipsychotic medication [5,9]. A patient's likelihood of adhering to prescribed medication is a product of an implicit and subjective assessment of the relative costs and benefits of adherence in relation to personal goals and constraints [3,9,11].

Recently, cessation of medication has been used as a measure of ineffectiveness in the management of schizophrenia [12-14]. The National Institute of Mental Health Clinical Antipsychotic Trials of Intervention Effectiveness (CATIE) schizophrenia trial was a large, randomized, controlled trial that evaluated the effectiveness of atypical and conventional antipsychotic medications in patients with schizophrenia over an 18-month period [14]. Its primary variable was the time taken to reach discontinuation of medication, for any reason. In this context, treatment discontinuation reflects in different proportions both patient and clinician views of efficacy and tolerability.

The significant impact of treatment adherence on clinical outcome and the increasing belief that continuation is as a proxy for overall effectiveness make it important for us to understand the reasons why treatment is in many cases discontinued. Randomized, controlled clinical trials may provide information that may help to shed light on what happens under clinical care. We, therefore, undertook a secondary analysis of actively-controlled trials of olanzapine for schizophrenia and schizophrenia-like illnesses to explore the reasons for treatment discontinuation by collapsing all the treatment groups. Our goal was to better understand the roles that efficacy and tolerability play in treatment discontinuation, along with the relative roles of patient and clinician perception.

## Methods

### Patient population

This was a post-hoc, pooled analysis of clinical trials within the Eli Lilly and Company database. The study selection criteria were 1) randomized, double-blind, active-controlled, 2) duration of 24 to 28 weeks, and 3) schizophrenia, schizophreniform disorder, or schizoaffective disorder. Four studies met these criteria. The 4 studies included 1627 patients treated with olanzapine, risperidone, quetiapine, or ziprasidone. None of the studies included a placebo arm. Patients were men and women between the ages of 18 and 75. All protocols were approved by the ethical review boards responsible for individual study sites. All patients gave written, informed consent prior to entering the study. The pooled analysis included 1 trial comparing olanzapine and risperidone [15], 1 trial comparing olanzapine and quetiapine [16], and 2 trials comparing olanzapine and ziprasidone [17,18]. Concomitant psychotropic medications were not allowed during the studies with the exception of limited benzodiazepines/hypnotics, approved antiparkinsonian medications, and in studies 2 and 4, antidepressants if a patient had been on a stable dose for 30 days prior to study enrollment and remained on a stable dose during the study.

### Study designs

Study 1 was a 28-week, multi-center study of olanzapine (10–20 mg/day, n = 172) versus risperidone (4–12 mg/day, n = 167) in inpatients and outpatients meeting diagnostic criteria for schizophrenia, schizophreniform disorder, or schizoaffective disorder according to the DSM-IV [15]. Patients had an initial score on the Brief Psychiatric Rating Scale (BPRS) of at least 42.

Study 2 was a 6-month, multicenter study comparing the efficacy of olanzapine (10–20 mg/day, n = 171) with quetiapine (300–700 mg/day, n = 175) in outpatients meeting DSM-IV criteria for schizophrenia and schizoaffective disorder who had poor functioning and prominent negative symptoms [16]. Patients had a score ≤60 on the Global Assessment of Functioning (GAF) and a score ≥4 on at least 3 or ≥5 on at least 2 of the 7 negative scale items on the Positive and Negative Syndrome Scale (PANSS).

Study 3 was a multicenter, 28-week study of olanzapine (10–20 mg/day, n = 277) and ziprasidone (80–160 mg/day, n = 271) in inpatients or outpatients meeting DSM-IV criteria for schizophrenia [17]. Patients had an initial score of at least 42 on the BPRS and a score ≥4 on 1 of the PANSS positive items in addition to a Clinical Global Impressions-Severity score ≥4.

Study 4 was a multicenter, 24-week, fixed-dose study of olanzapine (10, 15, or 20 mg/day, n = 202) and ziprasidone (80, 120, or 160 mg/day, n = 192) in inpatients or outpatients meeting DSM-IV criteria for schizophrenia or schizoaffective disorder with concurrent depressive symptoms [18]. Patients had a score ≥16 on the Montgomery-Åsberg Depression Rating Scale (MADRS) and ≥4 on Item 2 (reported sadness).

### Assessments

The primary objective of the present analysis was to assess the pattern and reasons for treatment discontinuation/continuation by pooling the 4 studies and collapsing all treatment groups. Clinical trial investigators in all 4 studies were required to record reason and date of discontinuation when patients left the trial before completing the study. A clinical report form with a checklist of potential reasons for discontinuation was used. The reasons for discontinuation are as follows:*1) Adverse Event (AE)-*with the event specified. *2) Entry Criteria Not Met*-checked when a patient had been inappropriately enrolled in the trial based on specific entry criteria. *3) Lack of Efficacy (LOE)-Patient Perception*-the patient perception was that symptom improvement was not adequate for continued use of the randomized medication. *4) Lack of Efficacy (LOE)-Physician Perception*-the physician perception was that symptom improvement was not adequate for continued use of the randomized medication. *5) Lack of Efficacy (LOE)-Patient and Physician Perception*. *6) Lost to Follow-up*-a patient did not come to a scheduled visit and subsequently was unable to be contacted by phone or mail. *7) Noncompliance*-patients intentionally missed all doses for a number of consecutive days specified for each trial or regularly took more than the prescribed amount of medication. *8) Personal Conflict*-the patient's decision for a variety of personal reasons, such as work conflict, lack of transportation, change of location, or unwillingness to fill out questionnaires. *9) Physician Decision*-physician decided that a patient should be discontinued due to reasons other than lack of efficacy or satisfactory response; examples include investigator sites closing and patients deemed unreliable. *10) Sponsor Decision*-the sponsor, Eli Lilly and Company, decided that a patient should be discontinued following consultation with the investigator treating the patient. *11) Clinical Relapse-Study 2 Only*-clinical relapse was based on predefined criteria, including an increase in the following positive symptoms of schizophrenia: delusions, conceptual disorganization, hallucinatory behavior, or suspiciousness as measured by PANSS; an increase in self depreciation as measured by the Calgary Depression Scale for Schizophrenia; or hospitalization for any psychiatric condition. *12) Satisfactory Response-Study 1 and Study 2 Only*.

For the present analysis, discontinuation due to lack of efficacy based on either patient or physician perception (reasons 3, 4, and 5) was grouped together and used as a measure of discontinuation due to poor symptom response to treatment (termed poor response). Discontinuation due to psychiatric adverse events (e.g., emergent psychosis or depression) along with "Clinical Relapse" (Study 2 only-reason 11) was used as a measure of discontinuation due to symptom worsening. These 2 categorizations, poor response and symptom worsening, represent a continuum of treatment inefficacy. In contrast, discontinuation due to non-psychiatric adverse events was considered discontinuation due to medication intolerability.

The psychopathology of schizophrenia was measured by visitwise analysis of mean total scores on the PANSS [19]. The PANSS is a 30-item scale that was designed to capture numerous symptoms of schizophrenia, including delusions, grandiosity, blunted affect, poor attention, and poor impulse control.

### Statistical methods

The analyses in this research were conducted combining all the treatment groups in the 4 clinical studies. The differences in PANSS total scores between study completers and discontinued patients were tested using Analysis of Variance (ANOVA) with term for group (completed vs. discontinued) at all timepoints that were common for all studies (Weeks 2, 4, 6, 8, 16, 20, and 24). In addition, PANSS total scores were also compared among completers and patients who discontinued due to various reasons, such as poor response/symptom worsening, intolerability to medication, and others using ANOVA with term for group. Logistic regression analysis was applied to test if early response predicts study completion with the independent variable as change in PANSS total at Week 2. A similar model was used with the predictor as a categorical variable defined as an improvement of 20% or greater in PANSS total from baseline to 2 weeks.

Data on treatment discontinuation due to poor response (lack of efficacy) were further assessed to compare the patient's role to the physician's role in the perception of treatment ineffectiveness and subsequent discontinuing medication. In order to emphasize patient attitude toward treatment response and the role of the patient in the decision to discontinue treatment, patient perception was based on discontinuation by patient perception of poor response either alone or in consensus with physician perception (reasons 3 and 5) for the purpose of the current analyses. Physician perception was based on physician perception alone (reason 4). It should be noted that results regarding perception would differ if patients that discontinued due to poor response based on the consensus of both patient and physician perception were categorized differently. Time to discontinuation due to poor response was assessed by Kaplan-Meier estimators for patient perception and physician perception. Clinical response as measured by PANSS total scores were compared between patient perception and physician perception at all available visits using ANOVA. For patients who discontinued due to adverse events, the actual event was identified for all but 6 patients. All statistical tests are based on a 2-tailed significance level of .05.

## Results

Table 1 summarizes the study sample at baseline across the 4 studies with treatment groups combined. A majority of patients were male (64.4%), Caucasian (53.3%), and had a diagnosis of schizophrenia (78.5%). The patient mean age was 39.5 ± 10.8 years, and the mean age of illness onset was 23.5 ± 8.3 years. The mean baseline PANSS total score was 91.27 ± 19.72, and 49.8% of patients had been hospitalized prior to the study.

**Table 1:** Patient and disease characteristics

Characteristic	Study 1 (n = 339)	Study 2 (n = 346)	Study 3 (n = 548)	Study 4 (n = 394)	**Total (N = 1627)**
Age (mean ± SD)	36.21 ± 10.73	41.05 ± 9.58	39.10 ± 11.8	41.59 ± 9.74	**39.53 ± 10.85**
Sex (Male %)	220(64.9)	228(65.9)	352(64.2)	248(62.9)	**1048(64.4)**
Race (Caucasian %)	253(74.6)	179(51.7)	239(43.6)	197(50.0)	**868(53.3)**
Diagnosis (%)					
Schizophrenia	277(81.7)	230(66.5)	548(100)	223(56.6)	**1278(78.5)**
Schizoaffective	52(15.3)	116(33.5)	--	171(43.4)	**339(20.84)**
Schizophreniform	10(2.9)	--	--	--	**10(0.6)**
Age of Onset Illness (yrs ± SD)	23.51 ± 7.48	23.36 ± 8.21	23.37 ± 8.27	23.71 ± 8.93	**23.48 ± 8.26**
PANSS Total (mean ± SD)	96.08 ± 16.55	84.83 ± 14.03	100.9 ± 20.18	79.35 ± 17.51	**91.27 ± 19.72**
Prior Hospitalization (%)	337(99.4)	180(52.0)	105(19.2)	189(48.0)	**811(49.8)**
Hospitalization Days (mean ± SD)*	23.12 ± 43.89	55.45 ± 77.13	16.10 ± 37.4	41.12 ± 44.10	**33.58 ± 54.31**
Illness Duration (yrs ± SD)	12.57 ± 9.75	17.68 ± 9.50	15.80 ± 11.63	17.84 ± 10.59	**16.02 ± 10.75**

* Mean hospitalization days for group of patients reporting prior hospitalization.

### Reasons for discontinuation

**Figure 1:**
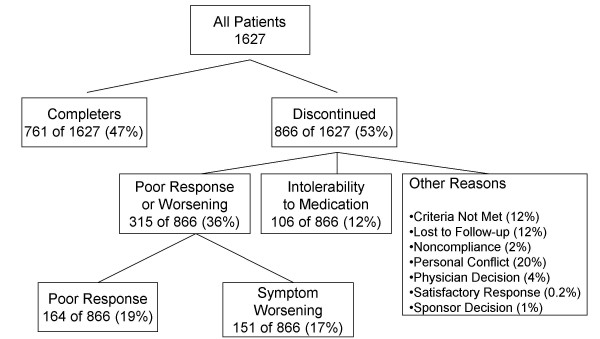
**Flowchart of early treatment discontinuation. **Values are the summary of reasons for discontinuation from all 4 studies. Poor Response was based on "Lack of Efficacy." Symptom Worsening was based on "Psychiatric Adverse Events" and *a priori *protocol defined "Clinical Relapse" (Study 2 only).

A majority of patients (53%; 866/1627) discontinued early from these 4 studies. The reasons for discontinuation are summarized in Figure 1. The most common reason for early treatment discontinuation was poor response/psychiatric symptom worsening (36%; 315/866), which was 3 times the rate of patient discontinuation due to medication intolerability (12%; 106/866). In particular, poor response accounted for 19% (164/866) of patient discontinuation, and symptom worsening accounted for 17% (151/866). The most common psychiatric adverse events (symptom worsening) cited for treatment discontinuation were psychosis (n = 35); suicide ideation, attempts, or completion (n = 18); schizophrenia, schizoaffective, or schizophreniform disorder (n = 17); and depression (n = 12). The non-psychiatric adverse events (medication intolerability) most frequently cited for treatment discontinuation were sedation (n = 7), somnolence (n = 7), abnormal ECG (n = 6), vomiting (n = 5), dizziness (n = 4), dystonia (n = 3), fatigue (n = 3), abnormal liver function test (n = 3), and increased weight (n = 3). The rates of and reasons for discontinuation across the 4 studies were fairly consistent and are shown in Table 2.

**Table 2:** Reasons for discontinuation by study

Reason for Discontinuation n (%)	Study 1 (n = 339)	Study 2 (n = 346)	Study 3 (n = 548)	Study 4 (n = 394)	**Total (N = 1627)**
Overall Discontinuation	161(47.5)	190(54.9)	268(48.9)	247(62.7)	**866(53.2)**

Poor Response or Worsening	67(41.6)	78(41.0)	99(36.9)	71(28.7)	**315(36.4)**
Intolerability to Medication	19(11.8)	16(8.4)	31(11.6)	40(16.2)	**106(12.2)**
Other Reasons*	75(46.6)	96(50.5)	138(51.5)	136(55.1)	**445(51.4)**

*Other reasons for discontinuation included criteria not met, lost to follow-up, noncompliance, personal conflict, physician decision, satisfactory response, and sponsor decision.

### Symptom response: Completers versus patients who discontinued

**Figure 2:**
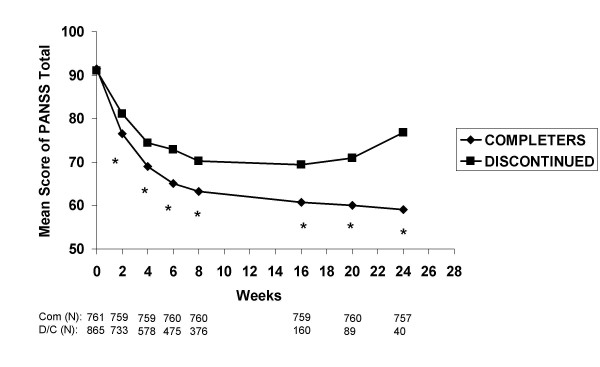
**Visitwise PANSS total scores between patients who completed the study and those who discontinued early. **Values are means across all treatments and studies. *p < .001 between group difference. Completers (Com), Discontinued (D/C).

In order to objectively examine the association between poor clinical outcome and treatment discontinuation independently of the checklist used at patient departure, PANSS total scores at each assessment were compared between patients who completed the study and those who discontinued early. There was no significant difference in baseline PANSS total scores between the patients who completed and those who discontinued treatment (91.4 ± 19.2 and 91.1 ± 20.2, respectively; p = .744). However, at each timepoint after treatment had begun, patients who completed the study had significantly lower PANSS total scores than patients who discontinued prior to the end of the study (p < .001, Figure 2). Although both groups showed significant clinical improvement compared to baseline (p < .001 at each timepoint), patients who discontinued early from the study appeared to have a slower initial rate of improvement and less improvement overall as compared to those patients who completed the study.

In view of these results, it was of interest to determine if early response predicted study completion. Symptom improvement from baseline to 2 weeks as measured by mean change in PANSS total score was significantly predictive of study completion (regression coefficient estimate 0.02, p < .001). Further, defining early response as a 20% or greater improvement in PANSS total score at 2 weeks, 28.9% of patients (431 of 1492 available at Week 2) met the criteria. Based on this criteria, early response was associated with an approximately 80% greater likelihood of completing the study (odds ratio 1.76, confidence interval 1.4, 2.21, p < .0001).

**Figure 3:**
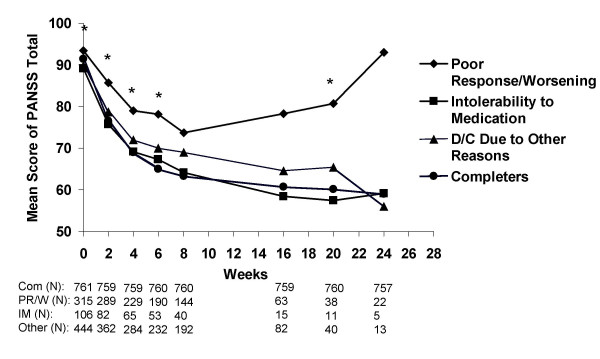
**Visitwise PANSS total scores between patients who completed the study and those who discontinued early due to various reasons. **Values are means across all treatments and studies. *p < .05 between group difference. Completers (Com), Poor response/symptom worsening (PR/W), Intolerability to Medication (IM).

PANSS total scores at each assessment were also compared among completers and patients who discontinued treatment early due to poor response/symptom worsening, intolerability to medication, and "other," respectively. "Other" reasons for discontinuation were the same as those described in Figure 1 and as described in detail in the Methods section. There was a cross-group significant difference from Week 0 through Week 6 and also at Week 20 (p < .05; Figure 3). In contrast to patients who discontinued due to poor response or symptom worsening, patients who discontinued due to medication intolerability showed improvement in PANSS total scores comparable to study completers, suggesting that adverse events do not interfere with symptom response but do prevent an otherwise effective treatment.

The baseline characteristics of patients who discontinued early due to poor response or symptom worsening are described in Table 3. This group of patients reported prior hospitalization significantly more frequently than all other patients (56.8%; 179/315 vs. 48.2%; 632/1312; p = .007). In addition, these patients were significantly more often Caucasian when compared with all other patients (64.4%; 203/315 vs. 50.7%; 665/1312; p < .001). Other baseline characteristics were similar between patients who discontinued due to poor response or symptom worsening and the rest of the study population.

**Table 3:** Baseline characteristics of patients who discontinued due to poor response or symptom worsening

Characteristic	Study 1 (n = 67)	Study 2 (n = 78)	Study 3 (n = 99)	Study 4 (n = 71)	**Total (N = 315)**
Age (mean ± SD)	36.17 ± 11.14	39.56 ± 9.36	38.58 ± 11.95	41.77 ± 9.91	**39.03 ± 10.84**
Sex (Male %)	43(64.2)	57(73.1)	58(58.6)	53(74.6)	**211(67.0)**
Race (Caucasian %)*	54(80.6)	48(61.5)	53(53.5)	48(67.6)	**203(64.4)**
Diagnosis (%)					
Schizophrenia	57(85.1)	54(69.2)	99(100)	39(54.9)	**249(79.8)**
Schizoaffective	8(11.9)	24(30.8)	--	32(45.1)	**64(20.5)**
Schizophreniform	2 (3.0)	--	--	--	**2 (0.6)**
Age of Onset Illness (yrs ± SD)	22.80 ± 6.69	21.63 ± 6.85	22.44 ± 7.62	22.63 ± 7.43	**22.36 ± 7.18**
PANSS Total (mean ± SD)	95.48 ± 15.31	86.60 ± 15.60	105.83 ± 22.04	82.01 ± 16.82	**93.50 ± 20.35**
Prior Hospitalization (%)*	65(97.0)	45(57.7)	27(27.3)	42(59.2)	**179(56.8)**
Hospitalization days (mean ± SD)^†^	35.75 ± 54.52	73.11 ± 113.67	24.96 ± 69.32	49.14 ± 50.12	**46.66 ± 76.40**
Illness Duration (yrs ± SD)	13.25 ± 10.47	17.93 ± 9.53	16.13 ± 12.02	19.13 ± 10.83	**16.65 ± 10.99**

*Significantly different from all other patients (prior hospitalization, p = .007; Caucasian race, p < .0001).

^† ^Mean hospitalization days for group of patients reporting prior hospitalization.

### Patient perception

**Figure 4:**
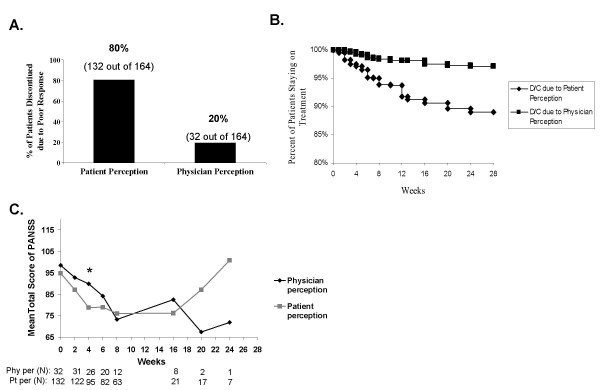
**Patient and physician perception of efficacy**. **A. **Discontinuation due to poor response by perception. Patient perception was based on discontinuation by patient perception of poor response either alone or in consensus with physician perception. Physician perception was based on physician conclusion alone. **B**. Time to discontinuation (D/C) due to poor response by perception. **C**. Visitwise PANSS total scores between patients who discontinued early due to poor response by patient perception and those by physician perception. *p < .05. Physician perception (Phy per), Patient perception (Pt per).

Discontinuation due to poor response was further characterized to determine whether the patient or physician concluded that symptom response was not adequate for medication continuation. In order to emphasize the role of patients in the decision to discontinue, discontinuation due to patient perception of poor response in the following analyses was defined as discontinuation due to patient perception of poor response (lack of efficacy) either alone or in consensus with physician conclusion. Physician perception was based on physician perception of poor response alone. Patient perception accounted for 80% (132/164) of discontinuation of treatment due to poor response (Table 4 and Figure 4A). In addition, the time to discontinuation was much sooner when patient perception in comparison to physician perception of poor response was the reason for discontinuation (Figure 4B). However, when PANSS total scores were compared between the groups that discontinued due to poor response by patient perception and by physician perception, the clinical performance was similar between the 2 groups (Figure 4C; the only significant difference in PANSS scores between the groups was at Week 4, p = .02).

**Table 4:** Discontinuation due to poor response by patient and/or physician perception

Reason for Discontinuation	Study 1	Study 2	Study 3	Study 4	**Total**
**Poor Response by Patient Perception Either Alone or in Consensus With Physician **n (%*)	40(76.9)	18(85.7)	46(80.7)	28(82.4)	**132(80.5)**
Poor Response by Patient Perception Alone n (%*)	11(21.2)	9(42.9)	25(43.9)	14(41.2)	**59(36.0)**
Poor Response by Patient and Physician Perception n (%*)	29(55.8)	9(42.9)	21(36.8)	14(41.2)	**73(44.5)**
**Poor Response by Physician Perception **n (%*)	12(23.1)	3(14.3)	11(19.3)	6(17.6)	**32(19.5)**

*Denominator is the total number of patients that discontinued due to poor symptom response.

## Discussion

Premature treatment discontinuation was very common in these 4 schizophrenia clinical trials. The most common reason for early treatment discontinuation was poor symptom response/psychiatric symptom worsening, which was substantially more common than discontinuation due to medication intolerability. Patients who discontinued early from the study due to poor response or symptom worsening had a slower initial rate of improvement and less improvement overall compared to those patients who completed the study. In contrast, patients who discontinued due to medication intolerability were showing symptom improvement comparable to study completers up until discontinuation. Discontinuation due to poor response was overwhelmingly linked to patient perception of response compared to physician observation alone. Discontinuation due to patient perception of poor response appeared to occur particularly early in the treatment course.

The substantial rate of premature discontinuation from these studies likely reflects the great challenge of treatment adherence in the clinical treatment of patients with schizophrenia. Discontinuation by more than half of the patients in these trials is within the broad range of previous reports of antipsychotic discontinuation (25%–75%) and is in line with reports of medication adherence in naturalistic settings [1-4]. Both the present study and other analyses of clinical trials likely underestimate the true incidence (and likely the associated burden) of antipsychotic nonadherence in the long-term course of therapy since the studies are time-limited, and treatment of schizophrenia is a life-long consideration. Studies of older typical antipsychotics indicate that about 75% of patients discontinue their medication within 2 years [5].

Perhaps the most interesting and important result of our analysis is that treatment discontinuation due to inadequate control of psychiatric symptoms appeared 3 times as likely as discontinuation due to medication intolerability. This was an unexpected finding of this systematic study since adverse events are a commonly cited reason for medication nonadherence [5,9]. Clinician experience with the older typical antipsychotics may be partially responsible for the perception of medication intolerability being a more common reason for antipsychotic treatment discontinuation. Other studies of patient attitudes toward antipsychotic treatment adherence suggest that adverse events may be important for patient attitudes toward antipsychotic adherence in particular with typical antipsychotics [20]. The transition of treatment of patients with schizophrenia and related disorders from typical to atypical agents over the past 10–15 years may have resulted in a decrease in the incidence of discontinuation due to adverse events relative to poor efficacy.

The high rate of discontinuation in the present study is consistent with the results of the large, 18-month CATIE trial in which 74% of patients discontinued their assigned antipsychotic medication [14]. Treatment discontinuation because of lack of efficacy (24%) was also more common in the CATIE trial than discontinuation because of medication intolerability (15%), consistent with the studies reported here.

Using an objective rating scale (PANSS), patients who discontinued had less improvement compared to study completers, suggesting that this patient group has a real deficit in medication response. However, even patients who discontinued achieved response to a certain extent, suggesting that there is a critical level of response needed to keep patients on treatment. Patients appeared to be especially likely to give up on treatments that did not provide a rapid therapeutic response, as a 20% early response resulted in an approximately 80% greater likelihood of remaining on therapy. Although the notion that a substantial delay in antipsychotic response is common in the field of psychiatry [21], Agid et al. [22] recently reported the results of a meta-analysis of double-blind, controlled trials that suggests antipsychotic response starts within the first week of therapy and accumulates over time. In addition, antipsychotic response within the first week of treatment has been reported to predict response after 6 weeks, at least for haloperidol [23]. The present study expands upon these findings by suggesting that early response also predicts treatment continuation. Therefore, patient perception of efficacy early on in treatment may be a major contributor to engagement in treatment and adherence to the treatment plan.

One possible explanation for the inadequate treatment response of patients who discontinued treatment early due to poor response or symptom worsening may be that they are intrinsically less responsive to treatment. For some patients, treatment resistance may represent an intrinsic part of the schizophrenic illness, at least with current antipsychotic medications [24]. The current study provides limited data to address this possibility. In support of this hypothesis, patients who discontinued treatment early due to poor response or symptom worsening reported prior hospitalization significantly more than all other patients. Treatment-resistant patients often require extensive periods of hospitalization, and older studies commonly used frequent hospitalization as an indicator of treatment resistance, although this may not be accurate in all cases [25]. On the other hand, other baseline characteristics that might be associated with treatment resistance, such as duration of illness and baseline illness severity, were similar between patients who discontinued due to poor response or symptom worsening and the rest of the study population. Standardized criteria for treatment resistance have been more recently described [26,27]. Further studies using defined criteria for treatment resistance are needed to better determine if patients who discontinue treatment due to poor response or symptom worsening are truly treatment resistant. Alternatively, these patients may discontinue early because of a suboptimal early treatment response and perceived lack of efficacy, and may possibly benefit from an early, active clinician-initiated attempt to bolster engagement in order to maintain treatment adherence.

Interestingly, patients who discontinued due to medication intolerability were showing symptom improvement comparable to study completers prior to their discontinuation. It should be noted that some of the adverse events were not considered severe; nonetheless, they were costly to patients by derailing patients from the potential benefit of the treatment. Had these patients continued their treatment to study completion, they would have likely had a clinically successful treatment. Therefore, adverse events should be viewed as a significant barrier to treatment efficacy and must be addressed on an individual patient basis.

Weight gain is a potential adverse event of atypical antipsychotic treatment that has been cited by patients as highly distressing [28] and has been proposed to be a factor in medication nonadherence [3]. However, weight gain has rarely been directly investigated as a factor in medication discontinuation or adherence. In the present studies, weight gain was infrequently reported as a reason for discontinuation (3 patients; 0.2%). However, weight gain in more naturalistic treatment settings may be a more important factor in patient adherence to medication than reported here for clinical trials. Consistent with this notion, in the CATIE trial, which was designed to have several "real-world" features in order to make the results more generalizable, there was a higher rate of treatment discontinuation due to weight gain than reported here [14]. Four percent of all patients discontinued due to weight gain or metabolic effects, although the incidence of discontinuation due to weight gain or metabolic effects was higher in olanzapine-treated patients (9%).

In addition to the deficit in treatment response as measured by PANSS scores, there was also a subjective component to the poor response reported by some patients. While patient perception of poor response was responsible for treatment discontinuation much more frequently than physician perception of poor response, patients who discontinued due to patient perception of poor response and patients who discontinued due to physician perception of poor response had similar PANSS scores, highlighting a subjective aspect of patient perception of treatment effectiveness. This suggests that patients are aware of whether they are getting better and may not be as willing as physicians to allow more time for symptom improvement if they perceive early in treatment that their symptom response is less than optimal. These results are consistent with the health belief model that suggests a patient's likelihood of adhering to prescribed medication is a product of an implicit and subjective assessment of the relative costs and benefits of adherence in relation to personal goals and constraints [3,9,11]. These results highlight the importance of active engagement of the patient early in treatment, with a clear understanding of the patient's expectations and treatment goals.

A limitation of the present analysis is that the reasons for discontinuation on the checklist used to categorize discontinuation in the 4 clinical trials may have not optimally captured the primary reason for discontinuation in all cases. For instance, a relatively high number of patients discontinued due to "personal conflicts," which included a variety of specific reasons and were not analyzed in this study. Categorizing all of these reasons as personal conflicts may have prevented the identification of additional barriers to medication adherence and may mask an underlying patient concern regarding efficacy, tolerability, or novel reasons that caused patients to lack the motivation to continue. Finally, discontinuation due to physician perception of response may have been underrepresented since physician versus patient perception was only captured in regards to poor symptom response and was not assessed in regards to symptom worsening (psychiatric adverse events). Although discontinuation due to psychiatric adverse events was ultimately a physician decision, it cannot be concluded that patients did not play a role in this decision. Therefore, discontinuation due to symptom worsening was not considered in the analysis of discontinuation due to patient and physician perception of response.

An additional limitation of the present study is that it is based on clinical trials that may not reflect more naturalistic patient treatment settings. Patients enrolled in the trials were much more homogeneous than the patient population seen in routine clinical care because of restrictions on patient enrollment. For instance, in the trials, patients with alcohol and substance dependence were excluded, and patients generally did not receive polypharmacy. In addition, clinical trials require that patients be motivated to participate in the trial, so these patients may have different implicit motivation and beliefs regarding treatment than patients in other settings. As a consequence, the rates of and reasons for discontinuation reported in this study may not be generalizable to typical outpatient settings. With these caveats, the systematic investigation of reasons for early discontinuation in the present study may still help to develop strategies to improve patient engagement in long-term therapy.

## Conclusion

In these studies, as in clinical management of patients with schizophrenia, treatment discontinuation was strikingly common. Poor response to treatment and worsening of underlying psychiatric symptoms, and to a lesser extent, intolerability of medication were the most common reasons for treatment discontinuation. Both a real inadequacy of treatment response, as well as patient perception of failure to improve, contributed to early treatment discontinuation. Improved treatment adherence in schizophrenia can reduce the risk of relapse and its morbid consequences, and perhaps promote higher functioning through better therapeutic engagement and by building upon improvement. This study suggests that adherence may be enhanced by effective symptom control as objectively measured, and as subjectively perceived.

## Competing interests

All authors are employees of Eli Lilly and Company.

## Authors' contributions

HLS made substantial contributions to the analysis design, data analysis, and critical revision of the manuscript. DHA made substantial contributions to the interpretation of data, drafting, and critical revisions to the manuscript. BJK made substantial contributions to the individual study designs, analysis design, and critical revisions to the manuscript. All authors read and approved the final manuscript.

## Pre-publication history

The pre-publication history for this paper can be accessed here:


